# Ubiquitous presence of pesticides in feathers of common UK birds

**DOI:** 10.1007/s11356-026-37677-0

**Published:** 2026-04-21

**Authors:** Priyesha Tank, Cannelle Tassin-de-Montaigu, Gaetan Glauser, Sylvie Guinchard, Dave Goulson

**Affiliations:** 1 University of Sussex School of Life Sciences, Brighton, UK; 2 Universite de Neuchatel Faculte Des Sciences, Neuchatel, Switzerland

**Keywords:** Insecticides, Herbicides, Neonicotinoid, Passerine, Permethrin, Clothianidin, Imidacloprid, Pendimethalin

## Abstract

Pesticides continue to be widely used despite the concerns about their negative impacts on non-target organisms. Birds are at risk from exposure to several classes of pesticides regardless of the habitats they are found in, since pesticides are applied in rural and urban areas. In some instances, the doses received have been found to cause lethal and/or sublethal effects. However, levels of pesticide exposure in wild birds remain largely unknown and this study aims to shed light on this matter. Feathers from five widespread bird species (blackbird *Turdus merula,* blue tit *Cyanistes caeruleus*, chaffinch *Fringilla coelebs*, dunnock *Prunella modularis* and goldfinch *Carduelis carduelis*) were collected from seven sites across the UK and Republic of Ireland from a variety of habitats. These were tested for the presence of 17 pesticides and 5 insecticide metabolites. All samples contained measurable levels of pesticides (> limit of quantification) and an average of four pesticides were present per sample. The most frequently occurring pesticides included the insecticides permethrin, chlorpyrifos, clothianidin and imidacloprid, and the herbicide pendimethalin. Permethrin was the most dominant pesticide present at all sites and in 98% of samples. These results highlight the prevalence of pesticide contamination using a non-intrusive method, which can be of value to monitor pesticide presence in the environment and flag up those of particular concern needing further investigation.

**Supplementary Information:**

The online version contains supplementary material available at 10.1007/s11356-026-37677-0.

## Introduction

Agrochemicals such as pesticides have become an integral component of the agricultural industry, contributing to significant increases in global food production since their introduction (Alexandratos and Bruinsma 2012). However, their use has also led to pesticide pollution and environmental contamination on a global scale which has contributed to biodiversity loss and disruption of many ecosystems (Köhler and Triebskorn 2013; Carvalho 2017; Rigal et al. 2023). Both wild and domestic animals are exposed to pesticides, as are humans. Multiple pesticide residues are commonly found in human tissue samples (Cruz et al. 2003; Bedi et al. 2013), and even the cerebral spinal fluid can be contaminated (Li et al. 2022). These issues have been recognized for a long time—the release of *Silent Spring* by Rachel Carson in 1962 already highlighted the damage caused by pesticides (Carson 1962), yet they remain largely unresolved.

Much recent research on the impacts of pesticides on wildlife has focussed on pollinators, particularly bees (Sanchez-Bayo and Goka 2014). Avian species have received less attention. UK farmland specialist bird species have decreased by 90% between 1970 and 2018 (Massimino et al. 2022), and many are now classified as vulnerable or endangered on the International Union for Conservation of Nature red-list. Farmland bird decline is also evident across Europe and North America where pesticide use is considered to be a significant factor negatively affecting their numbers (Stanton et al. 2018; Li et al. 2020; Massimino et al. 2022; Rigal et al. 2023; Molenaar et al. 2024).

Pesticides can affect birds through both indirect and direct pathways. Indirectly, pesticide use reduces the availability of insect prey and weed seeds, which can lower breeding success and survival. Direct effects occur when pesticides are toxic to birds themselves, impairing fitness or causing mortality. A well-known historical example is the bioaccumulation of DDT in avian fatty tissues, which caused eggshell thinning and significant population declines in predatory bird species (Porter and Wiemeyer 1969; Olsen et al. 1993). Although this case is now largely historic, it represents a pivotal moment in avian ecotoxicology.

More recent studies demonstrate how modern pesticides can also exert direct sublethal effects on birds. Ingestion of seeds treated with imidacloprid, a neonicotinoid insecticide, caused sublethal impacts in white-crowned sparrows *Zonotrichia leucophrys*. Although birds are less sensitive to neonicotinoids than insects, these compounds affect similar neural pathways, leading to impaired migration, disorientation and reduced foraging and feeding efficiency (Eng et al. 2017). Experimental work by Gaffard et al. (2022) further demonstrated that feeding birds conventionally grown grains containing detectable pesticide residues resulted in sublethal effects, including reductions in offspring quality, relative to birds fed organically farmed grain. Neonicotinoids have also been shown to induce physiological stress and negatively affect reproductive outcomes across multiple avian species (Molenaar et al. 2024). Similarly, chronic exposure to the fungicide tebuconazole in house sparrows *Passer domesticus* resulted in adverse effects on offspring physiology and reduced survival (Bellot et al. 2025).

Experimental exposure studies conducted in academic laboratory settings tend to focus on effects of single pesticides, but in the field, individuals are likely to be chronically exposed to pesticides that are applied simultaneously via multiple routes, creating a cocktail effect. It is challenging to encompass these factors; however, it is important to keep this in consideration when conducting scientific exposure studies so that they are as relevant and realistic as possible. Pesticide mixtures can have additive or synergistic effects on individuals and further impact survival and breeding success. For example, studies on the red avadavat *Amandava amandava* have found that exposure to a mixture of mancozeb (dithiocarbamate fungicide) and imidacloprid (neonicotinoid insecticide) disrupted the thyroid gland which can impact reproductive development (Pandey and Mohanty 2017).

Birds can be exposed to pesticides in several ways, including consumption of contaminated seeds/plants or insect prey (terrestrial and aquatic), drinking contaminated water, inhalation of aerosols or dust, or topical exposure. For example, fipronil and neonicotinoid insecticides have been commonly found in rivers and waterways (Hallmann et al. 2014; Perkins et al. 2021). In these circumstances, it is highly likely that birds are ingesting pesticides from drinking water, consuming contaminated aquatic insects and/or are exposed to contaminated water during bathing.

Screening feathers for pesticides provides an indication of exposure to banned and legacy pesticides such as DDT and neonicotinoids (Dauwe et al. 2005; Klaas‐Fábregas et al. 2024; Bouso et al. 2025). Many pesticides accumulate in the nestling phase most likely caused by exposure to contaminated nesting material (Perkins et al. 2021; Tassin de Montaigu et al. 2025) and any pesticides that have entered the bloodstream will accumulate internally in feathers while growing. Therefore, internal contamination in feathers can provide a snapshot of environmental contamination prior to and during the growth phase. Meanwhile, external contamination may accumulate over time and can change throughout the growth phase to the moulting stage. Measuring each can be used to understand historic and more recent levels of pesticide contamination in the environment. Humann-Guilleminot et al. (2023) have shown that clothianidin (neonicotinoid pesticide) accumulates in bird feathers following dietary exposure to field-relevant doses. It should also be noted that in some birds, moulting is a means to excrete mercury accumulation (Pedro et al. 2015), and this could also be true for other contaminants such as pesticides.

Neonicotinoid insecticides have been detected on house sparrow feathers collected across conventional and organic farmland in Switzerland (Humann-Guilleminot et al. 2019). Neonicotinoid insecticides were detected in 100% of feather samples from individuals collected from both types of farms despite these pesticides not being used in organic farmland.

This raises the question of whether pesticides are also present in feathers collected from individuals from non-farmland habitats, where pesticides are applied in a limited manner, if at all. Thus, in this study, we tested this hypothesis using blackbird, blue tit, chaffinch, dunnock, and goldfinch feathers to identify and quantify pesticide presence. The aims of this study were to assess pesticide levels in feathers collected from wild birds and to discuss the potential sources of contamination.

## Methods and materials

### Species selection

Fifteen species were initially selected. These species were chosen as they are the most common species across the UK and most often caught by trained ringers as part of The British and Irish Ringing Scheme organised by the British Trust for Ornithology (BTO) (Woodward et al. 2020). By utilising trained volunteers that are a part of this scheme we maximised the potential number of participants and potential number of samples collected. This non-invasive method reduces harm to birds whilst also maximising the number of volunteers that can participate in sample collection as additional training or permits are not required. As bird ringing was limited due to lockdowns in place during the Covid-19 pandemic, the numbers of volunteers taking part and thus the number of samples that were received were lower than expected. Therefore, we could not test for temporal variations in the levels of pesticides in the feathers.

### Feather collection

Volunteer ringers were provided with instructions for feather collection. Volunteers were asked to collect discarded feathers only that were found in the ringing bag, in hands and/or on the ground which could be clearly assigned to an individual bird. Feathers were not plucked. We requested volunteers to collect all feather types possible; feather types we received were mostly body feathers but also many wing feathers. The rachis of the feather was removed during analysis as it did not grind well. Feathers from different individuals were kept separate and it was emphasised that volunteers only used one bag per individual. Samples were collected between January 2020 and August 2021. Prior to posting samples, volunteers were asked to store feathers in a cool and dry place. On receiving the samples at the university, the samples were stored in a −90 °C freezer until analysed. The ring number and date of collection were noted for each sample and stored individually. The ring numbers were used to collect biometric data available on the BTO Ringing Scheme database. A total of 511 samples were collected from 15 sites across the UK and Republic of Ireland (ROI) from 21 different species. The ringing sites were from a variety of habitats (Table 1).

**Table 1:** Sites from where individuals were caught during ringing and samples were collected. Second column lists their associated habitats according to BTO habitat classifications followed by the number of samples collected and the number of pesticides detected per site and the mean number of pesticides per sample

Site	Habitat	No. of samples collected	No. of pesticides detected	Mean number of pesticides per sample
Galway	Rural human site Improved grassland on farmland	3	8	5.3
East Sussex	Reed swamp Other farming	11	10	4.9
Kent	Scrubland Dry grassland	21	11	5.6
Leicestershire	Rural human site New plantation	21	14	6.1
Powys	Rural human site	2	5	4.5
Scottish Borders	Suburban	12	12	5.8
South Gloucestershire	Rural human site Reed swamp Broadleaved water-logged woodland	4	7	6.3

### Sample preparation

All feathers were weighed and measured. A minimum of 25 mg (± 2.5 mg) was required per sample to enable accurate quantification of residues. Across all birds, 511 feathers were collected. Chemical analyses were conducted on a subset of 94 feathers because the mass of several samples was too low. Fifty feathers were processed and analysed individually. The remaining 54 feathers were combined into 24 pooled samples, with each pool consisting of 2–4 feathers from the same species and ringing site to ensure sufficient mass for analysis. Thus, our final sample size was 74. Unfortunately, the low number of individual samples could not allow us to wash a small number of feathers and still have a viable sample size, therefore we were unable to compare internal and external pesticide contamination. Feathers analysed were from the following five species: blackbird *Turdus merula*, blue tit *Cyanistes caeruleus*, chaffinch *Fringilla coelebs*, dunnock *Prunella modularis* and goldfinch C*arduelis carduelis*. The pesticides tested can be found in Table 3. Samples were analysed using UHPLC-MS/MS to quantify the levels of pesticides present (see details below).

### Pesticide selection

Pesticides were selected based on evidence of avian toxicity, likelihood of exposure in UK arable farmland and analytical feasibility. A protocol to test for chlorpyrifos, fipronil and its main metabolites, flupyradifurone, all neonicotinoids, neonicotinoid metabolites and sulfoxaflor had already been established; these pesticides were prioritised. In addition to these, cypermethrin, deltamethrin, permethrin, pendimethalin, ivermectin and prothioconazole were added to the list of pesticides. These were selected based on evidence that they pose a risk to birds in the UK, since they are all moderately to highly toxic to birds and are applied in large quantities in UK arable farmland (Tassin de Montaigu and Goulson 2020). While additional pesticides were initially tested, the methods did not accommodate all the pesticides we aimed to analyse.

A wider range of pesticides were initially considered; however, practical and financial constraints limited the number of compounds in the final list. Feather samples were limited and the amount of material restricted us to a single extraction method. Consequently, we prioritised neonicotinoids and pesticides with similar properties. Future work should prioritise underrepresented pesticide groups such as herbicides and fungicides that are applied in high volumes and widely used in agricultural areas, and for which the effect on birds remains largely unknown.

### Chemical analysis

#### Chemicals

Solvents and additives for UHPLC-MS/MS were milli-Q water, LC-MS grade methanol from Biosolve Chimie, France, ULC-MS grade acetic acid and ammonium acetate from Biosolve. For sample preparation, we used milli-Q water and HPLC grade acetonitrile from Fisher Chemicals. The salts used for the QuEChERS were all from Sigma-Aldrich, Darmstadt, Germany. The C18 sorbent was from ZeoChem. Thiamethoxam, clothianidin, imidacloprid, imidacloprid-olefin, desnitroimidacloprid, acetamiprid, desmethylacetamiprid, thiacloprid, dinotefuran, nitenpyram, flupyradifurone, fipronil-sulfone, cypermethrin, deltamethrin, permethrin, ivermectin, pendimethalin and prothioconazole standards were obtained from Sigma-Aldrich. Sulfoxaflor was obtained from Clearsynth, Brampton, Ontario, Canada. Fipronil and chlorpyrifos were from Toronto Research Chemicals, Ontario, Canada, and fipronil-sulfide from Honeywell, Morris Plains, New Jersey, USA. Thiamethoxam-d_3_, clothianidin-d_3_, imidacloprid-d_4_, acetamiprid-d_3_ and thiacloprid-d_4_ were purchased from CDN Isotopes, Pointe-Claire, Quebec, Canada. Dinotefuran-d_3_ was purchased at EQ Laboratories GmbH, Augsburg, Germany. Nitenpyram-13C-d_3_ was obtained from Alsachim, Illkirch-Graffenstaden, France. Fipronil-^13^C_3_, cypermethrin-d_5_, deltamethrin-d_5_, and permethrin-d_5_ were from Sigma-Aldrich. Chlorpyrifos-d_10_, pendimethalin-d_5_ and ivermectin-d_2_ were from Toronto Research Chemicals.

#### Sample extraction

The preparation of feather samples was adapted from previous studies (Humann-Guilleminot et al. 2019; Bonmatin et al. 2021). Feathers were finely cut, and *ca*. 25 mg of material was placed in a 2 mL microcentrifuge tube. Two 5.6 mm stainless steel UFO beads were added, and the samples were ground for 8 min in a Retsch MM400 tissue lyser at 27 Hz. One millilitre of acetonitrile and 25 µL of internal standard solution containing isotopically labelled pesticides (in acetonitrile (40 ng/mL for thiamethoxam-d_3_, clothianidin-d_3_, acetamiprid-d_3_, thiacloprid-d_4_, dinotefuran-d_3,_ nitenpyram-13C-d_3,_ fipronil-^13^C_3_, cypermethrin-d_5_, deltamethrin-d_5_, and permethrin-d_5_, 8 ng/mL for imidacloprid-d_4_, 80 ng/mL for ivermectin-d_2_ and 2 ng/mL for pendimethalin-d_5_) were added, and the tube was shaken in the tissue lyser for 4 min at 30 Hz. After centrifugation, the supernatant was transferred to a 5 mL Eppendorf tube containing 1 g of a QuEChERS salt mixture composed of 0.615 g of MgSO_4_, 0.154 g of NaCl, 0.154 g of sodium citrate dihydrate and 0.077 g of sodium citrate sesquihydrate (Kammoun et al. 2019). The feathers pellet was re-extracted with 700 µL of acetonitrile and centrifuged, and the two supernatants were combined in the 5 mL Eppendorf tube containing the salts in excess. A volume of 1.6 mL of water was added, and the tube was vigorously shaken by hand for approximately 1 min. The tube was centrifuged at 6000 rpm for 5 min, and the upper phase was collected and placed in a 2 mL tube containing 45 mg of MgSO_4_ and 35 mg of C18 sorbent for dispersive solid-phase extraction. The tube was shaken for about 30 s, centrifuged, and the supernatant was transferred to a new 2 mL tube. The tube was then partially evaporated in a centrifugal evaporator at 5 °C until a residual volume of 50–100 µL remained. One hundred microlitres of 50% acetonitrile was added; the tube was vortexed for 10 s, ultrasonicated for 1 min and centrifuged, and the supernatant was transferred to a 0.2 mL PCR tube and centrifuged again. The supernatant was finally placed into an HPLC vial fitted with a conical insert and stored at −20 °C until analysis.

#### Sample analysis

Seventeen pesticides and five of their metabolites (Table 3) were analysed by ultra-high pressure liquid chromatography-tandem mass spectrometry (UHPLC-MS/MS) using an Acquity UPLC I-Class (Waters) coupled to a TQ-XS triple quadrupole mass spectrometer (Waters). The separation was performed at a flow rate of 0.4 mL/min on a Kinetex PFP column (75 mm × 2.1 mm i.d., 2.6 µM particle size) from Phenomenex maintained at 55 °C. Mobile phases were H_2_O + 0.05% acetic acid and 1 mm ammonium acetate (A) and methanol (B). The following gradient programme was used: 2–100% B in 7 min, holding at 100% B for 1.5 min, and re-equilibration at 2% B for 2.5 min. The injection volume was 2 µL. The triple quadrupole was operated in both positive and negative electrospray ionisation using fast polarity switching, and the multiple reaction monitoring (MRM) mode was used. Source parameters were as follows: capillary voltage + 3 kV/−1 kV, source temperature 150 °C, desolvation gas flow and temperature 1000 L/h and 450 °C, respectively, and cone gas flow 350 L/h. The whole system was controlled by Masslynx 4.2 (Waters), and the data were processed in TargetLynx (Waters). For each batch of samples (60–80 samples), a freshly prepared 6-point calibration curve (0.01, 0.05, 0.1, 1, 5 and 20 ng/mL) and two blanks were prepared, and a quality control (QC) standard (1 ng/mL) was run every 20 samples. In addition, analysis of blank feathers from swans collected from Neuchâtel Lake, Switzerland, and spiked at 10 ng/mL (*n* = 6), yielded recoveries between 80 and 120% for all compounds but prothioconazole, for which recovery was 67%. This provided accuracies between 80 and 120% and coefficients of variation below 15%. The method’s limits of detection (LOD) ranged from 0.03 to 2.67 ng/g, while the method’s limits of quantification (LOQs) ranged between 0.08 and 8 ng/g. The specific LODs and LOQs for each compound are provided in supplementary information (SI) Table.

### Statistical analysis

An ANOVA was conducted to test for differences in the number of pesticides present between sites and species. This test was repeated to examine differences in pesticide concentrations present between sites and species.

We also tested whether variations in pesticide concentrations may be associated with biometric measurements such as age, weight, wing length and sex. To test this, we conducted linear regression models using the package lme4 (Bates et al. 2015), where these parameters were set as independent variables and the total pesticide concentration detected as dependent. Extracted *p*-value relationships were statistically significant if extracted *p*-values were less than 0.05. We also conducted non-parametric tests to understand if pesticide concentrations varied significantly between different sexes (sex could not be determined for all individuals; the sex for these individuals has been labelled as unknown). For this, a Kruskal-Wallis test was conducted, a *p*-value was extracted, and a significance level of 0.05 was selected to conclude whether there is a statistically significant difference in pesticide concentrations among different sex groups.

## Results

Feather samples were collected from numerous sites across the UK and ROI via the BTO Ringing Scheme and 74 samples were analysed, from five species and 103 individuals across seven sites (of which 53 individuals were pooled in 24 samples). Habitats varied across all sites (Table 1 and Table 2).

**Table 2:** Samples per species. All samples analysed were from one of the listed species in the first column. Total samples combine both individual and pooled samples, individual samples are from a single individual, pooled samples combine between two to four individual samples, followed by the total number of pesticides detected in samples from the corresponding species and the mean number of pesticides per sample

	Number of samples				
Species	Total	Individual	Pooled	No. of pesticides detected	Mean number of pesticides per sample
Blackbird *Turdus merula*	55	40	15	16	5.7
Blue tit *Cyanistes caeruleus*	5	4	1	8	5.3
Chaffinch *Fringilla coelebs*	10	3	7	12	5.7
Dunnock *Prunella modularis*	2	2	0	6	5.0
Goldfinch *Cardeulis cardeulis*	2	1	1	9	6.0

All samples contained measurable levels of pesticides (concentrations equal to or above LOQ for the specific pesticide). The number of pesticides present in samples ranged between 3 and 11, with a mean of 5.64 pesticides per sample. Pesticide concentrations ranged from 3.54 to 166.09 ng/g with a mean concentration of 18.16 ng/g

**Fig. 1:**
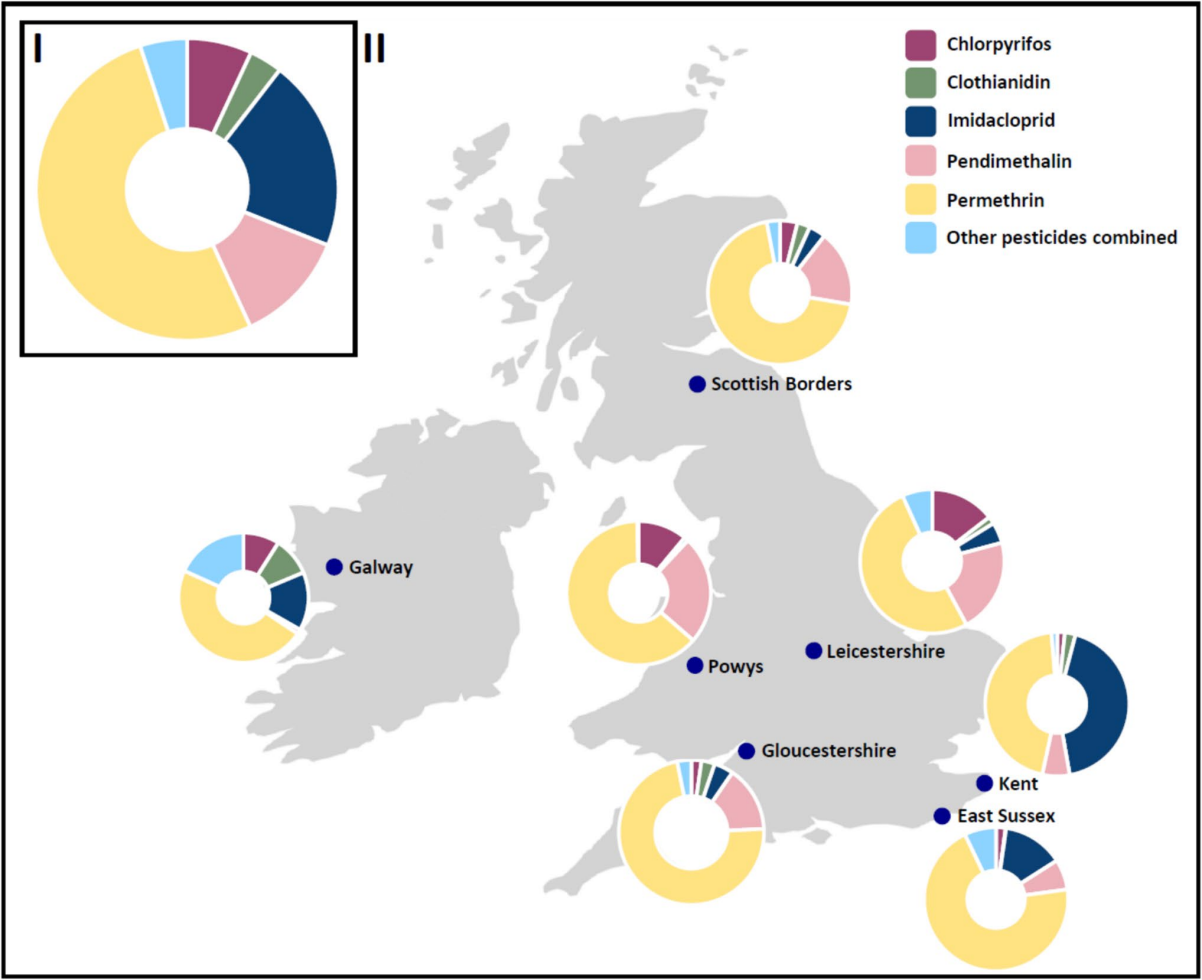
The relative proportion of overall concentrations of the five highest occurring pesticides. I. Concentration for all sites in the UK and ROI. II. Points for each ringing site where feathers were collected alongside corresponding pie charts

Permethrin was present in the greatest number of samples (97%), followed by chlorpyrifos (96%), pendimethalin (89%), imidacloprid (88%) and fipronil (72%) (Table 2). Summarised results for each pesticide are presented in Table 3. Similar patterns emerge with regard to the concentrations of different pesticides detected, with permethrin comprising 52% of the total pesticide concentration across all samples, followed by imidacloprid (21%) and pendimethalin (12%; Fig. 1).

There was a strong positive correlation (*R*^*2*^ = 0.78) between the number of samples and the total number of pesticides found at a site. However, sampling was uneven across the sites, and therefore it is likely that the difference in pesticides detected between sites was an artefact of sampling density.

The greatest number of samples were from sites in Kent and Leicestershire (21 samples each per site; including pooled samples) where 11 and 14 pesticides respectively were present in the samples tested from these sites. In contrast, only five pesticides were detected in samples collected in Powys where two samples were tested (Table 1). There was no difference in the mean number of pesticides present between sites (one-way ANOVA test: *F*-value = 1.27, *df* = 6, *p* = 0.281) or species (one-way ANOVA test: *F*-value = 0.23, *df* = 4, *p* = 0.921; SI Table 2). There was also no difference in the total concentration between sites (one-way ANOVA test: *F*-value = 1.05, *df* = 6, *p* = 0.404) or species (one-way ANOVA test: *F*-value = 0.53, *df* = 4, *p* = 0.717; SI Table 3).

**Table 3:** Summary statistics: Pesticides listed are those tested for in the analysis alongside their corresponding class and substance group. LOQ is the limit of quantification, and concentrations are given in ng/g. Abbreviations: *NN* neonicotinoid, *PP* phenylpyrazole

Pesticide	Pesticide class	Pesticide substance group	Samples > LOQ	% Samples > LOQ	Maximum (ng.g)	Median (ng.g)	Mean (ng/g)	SE
Acetamiprid	Insecticide	NN insecticide	4	5.41	0.19	0	0.01	0
Chlorpyrifos	Insecticide	Organophosphate insecticide	72	97.30	17.55	1.26	1.26	0.15
Clothianidin	Insecticide	NN insecticide	23	31.08	23.33	0.64	0.64	0.07
Cypermethrin	Insecticide	Pyrethroid insecticide	1	1.35	16.37	0.22	0.22	0.03
Deltamethrin	Insecticide	Pyrethroid insecticide	0	0	0	0	0	0
Desmethyl-acetamiprid *	Insecticide metabolite	NN insecticide metabolite	0	0	0	0	0	0
Desnitroimidacloprid	Insecticide metabolite	NN insecticide metabolite	0	0	0	0	0	0
Dinotefuran	Insecticide	NN insecticide	0	0	0	0	0	0
Fipronil	Insecticide metabolite	Phenylpyrazole insecticide	53	71.62	3.24	0.32	0.32	0.03
Fipronil sulfide	Insecticide metabolite	PP insecticide metabolite	2	2.70	0.05	0	0	0
Fipronil sulfone	Insecticide	PP insecticide metabolite	25	33.78	2.63	0.09	0.09	0.01
Flupyradifurone	Insecticide	Butenolide insecticide	7	9.46	4.97	0.14	0.14	0.02
Imidacloprid	Insecticide	NN insecticide	65	87.84	157.29	3.73	3.73	0.43
Imidacloprid-olefin	Insecticide	NN insecticide metabolite	5	6.76	0.71	0.03	0.03	0
Ivermectin	Veterinary medicine	Avermectin anti-parasitic	2	2.70	0.53	0.01	0.01	0
Nitenpyram	Insecticide	NN insecticide	0	0	0	0	0	0
Pendimethalin	Herbicide	Dinitroaniline herbicide	66	89.19	15.27	2.29	2.29	0.26
Permethrin	Insecticide	Pyrethroid insecticide	73	98.65	59.40	9.41	9.41	1.09
Prothioconazole	Fungicide	Triazole fungicide	0	0	0	0	0	0
Sulfloxaflor	Insecticide	Sulfoxamine insecticide	2	2.70	0.73	0.01	0.01	0
Thiacloprid	Insecticide	NN insecticide	11	14.86	1.47	0.04	0.04	0.01
Thiamethoxam	Insecticide	NN insecticide	7	9.46	3.03	0	0.06	0.01

### Biometric data and pesticide levels

**Fig. 2:**
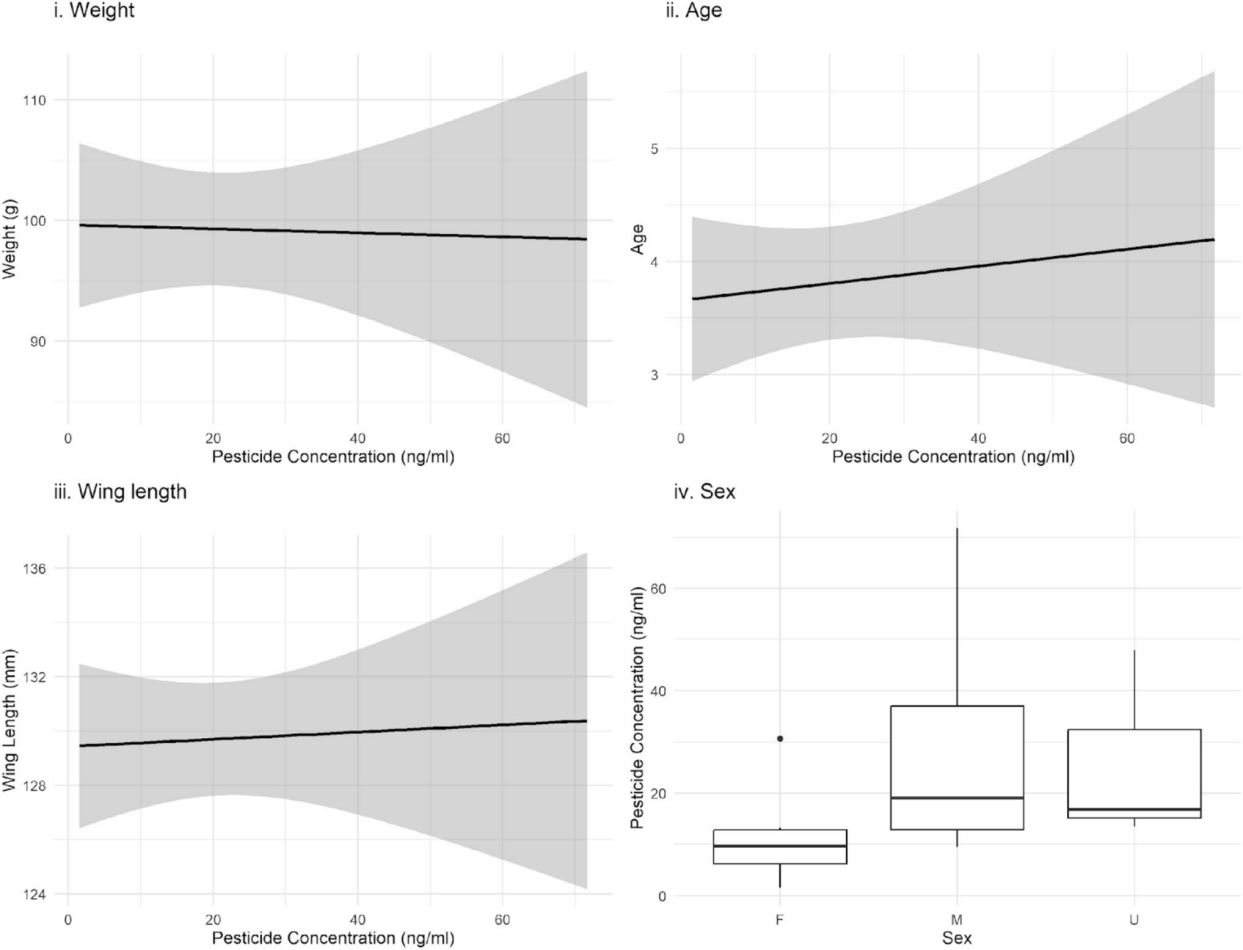
Relationships between biometric data (i) weight, (ii) age, (iii) wing length and (iv) sex (F: female, M: male and U: unknown) and total pesticide concentration detected in feather samples collected from blackbirds, sample size = 40

There was no statistically significant relationship (Fig. 2, R^2^ = 0.030, *p-*value = 0.943) between pesticide concentrations and the biometric parameters tested.

## Discussion

A range of pesticides and pesticide metabolites were detected in the bird feathers that were analysed from all the sites, regardless of habitat type or bird species. This demonstrates the ubiquitous presence of pesticides in the environment across the UK and Republic of Ireland.

The dominant pesticide, permethrin, was detected at all sites and in almost 99% of samples. It is unlikely that the contamination detected in the feathers analysed here can be explained by arable farmland use as only an estimated 114 kg of permethrin was applied between 2000 and 2015 across 3725 hectares in the UK (representing just 0.1% of the total arable area in the UK; Defra, 2025). However, this pyrethroid insecticide is also applied on livestock as an antiparasitic veterinary drug and is also commonly found in flea treatment drugs for dogs (Fernandez et al*.* 2011). Permethrin is likely to contaminate waterways when dogs are washed or enter rivers or streams when outdoors.

Several neonicotinoid insecticides were found at high frequency in the feather samples. These insecticides are ubiquitously found in the environment, from bird feathers to human plasma and urine (Humann-Guilleminot et al. 2019; Wood et al. 2019; Li et al. 2022). Here, we tested for seven neonicotinoids (acetamiprid, clothianidin, dinotefuran, imidacloprid, nitenpyram, thiacloprid and thiamethoxam) and three toxic metabolites (desmethyl-acetamiprid, desnitroimidacloprid and imidacloprid-olefin). Of these neonicotinoid insecticides, clothianidin, imidacloprid and thiamethoxam have been banned from use on field crops across the EU since 2018 (although some small-scale derogations have been granted). Acetamiprid is currently the only neonicotinoid authorised for agricultural use in the UK, where it is applied as a seed treatment for cereals or as a foliar spray on fruit (Pesticide Action Network UK, 2022). It is also found in some garden pest control products. However, it was found in just four of the feather samples, whereas the three neonicotinoids banned from outdoor agricultural use were found more often, and samples from all sites tested positive for at least one of them. Imidacloprid in particular was found in 88% of samples.

Imidacloprid contamination in the environment may originate from agricultural use, but imidacloprid was largely replaced with the newer neonicotinoids such as clothianidin and thiamethoxam several years before the 2018 ban (Defra, 2025). Recent studies have shown that veterinary medicinal products containing imidacloprid or fipronil (a neurotoxic insecticide not classed as a neonicotinoid, and the fifth most prevalent pesticide in our feather samples) may be significant contributors to environmental contamination even after being banned in agriculture (Perkins et al. 2021). Antiparasitic veterinary products are often applied to household pets to protect them from fleas and ticks, and these can enter waterways via household drains or when animals swim. One hundred percent of English rivers were positive for fipronil and its metabolites, and 70% were also contaminated with imidacloprid, both at levels that indicate high environmental risk to aquatic ecosystems (Perkins et al. 2021). Concentrations were significantly higher below wastewater treatment works, leading to the conclusion that use of these two pesticides as flea treatments on companion animals was the most likely source of river contamination.

Chlorpyrifos is another banned pesticide that has been detected in the tested feathers. This organophosphate inhibits acetylcholinesterase which causes the nervous system to dysfunction and causes paralysis and death in insects. This pesticide has also been found to be highly toxic to aquatic organisms, birds and humans which led to the EU ban in 2020. Despite the ban, chlorpyrifos was detected in samples collected after this period. It has also been detected in freshwater samples collected in the UK (Environment Agency, 2024). This is concerning as the threat of its effects remains long after its initial application and contaminates protected natural areas via waterways. This route of contamination can also impact birds that come into contact with these freshwater systems or connected environments which can occur in the form of bathing or drinking from these areas.

Another concerning result is that sulfoxaflor and flupyradifurone have also been detected at three sites in blackbird feathers (flupyradifurone in East Sussex and Leicestershire; sulfoxaflor in the Scottish Borders). Although labelled as butenolide and sulfoxamine insecticides, respectively, these compounds have the same mode of action as neonicotinoids (agonists of nicotinic acetylcholine receptors), which is how most pesticides are categorised (Cutler et al. 2013; Nauen et al. 2015). Sometimes known as *next generation neonicotinoids*, these compounds have been approved for use in the UK, but their use remains restricted. Sulfoxaflor is only approved for professional use and application is restricted to glasshouses or indoor use. Sulfoxaflor was detected in two feather samples, contamination that may have occurred because sulfoxaflor has leached into watercourses that are close to glasshouses where it has been applied. There is evidence for greenhouses contributing to pesticide occurrences in surface waters (Roseth and Haarstad 2010; Boye et al. 2022), but it is often difficult to pinpoint the source of contamination. Meanwhile, flupyradifurone occurrences are likely due to amateur use in homes and gardens, as it is approved for domestic use to control insect pests in the UK. Although in this study, these pesticides were only detected at low levels (maximum concentrations for flupyradifurone: 4.97 and sulfoxaflor: 0.73 ng/g), that they were detected in environmental samples at all is concerning given their very restricted use and high toxicity to insect life.

We also detected the herbicide pendimethalin at high prevalence (89% of samples). It is applied as a pre- and post-emergent herbicide to destroy and prevent growth of certain plants in arable regions and is also available for use in residential areas. In arable regions across the UK, its use has increased by 297% over 27 years (1990–2016) and in 2020, 866 tonnes were applied (Tassin de Montaigu and Goulson 2020; Defra, 2025). Despite herbicides being the most used pesticide class worldwide, they often receive less attention than insecticides when impacts of pesticides on the environment are debated. Pendimethalin and other herbicide residues have been found in honeybees, earthworms and insect boluses fed to nestlings (Cech et al. 2022). This herbicide is a risk to avian species because of its high usage in arable farmland alongside its relatively high toxicity to birds (Millot et al. 2015; Tassin de Montaigu and Goulson 2020). These findings suggest that pendimethalin requires further investigation with regard to the risk it poses to birds (Tassin de Montaigu and Goulson 2020; Cech et al. 2022).

Here we tested for 17 pesticides and five pesticide metabolites and found that a single feather sample weighing approximately 25 mg contained up to nine of these compounds. There are currently hundreds of pesticides in use across the UK and even more worldwide. Testing for a larger number of pesticides most likely would have led to many more being detected in the samples. Cocktails of pesticide residues have been detected in rivers, non-target organisms and soil (Miller et al. 2017; Casado et al. 2019; Tang and Maggi 2021). As we found here, residues of banned or discontinued pesticides are frequently detected. The effects of chronic exposure to complex cocktails of pesticides are very poorly understood. Most pesticide cocktails have additive effects; however, some pesticides are known to act synergistically such as compounds from the -azole group of pesticides. For example, combining propiconazole with flupyradifurone amplified the negative effects of the insecticide alone in honeybees *Apis mellifera* (Tosi and Nieh 2019). Similar synergistic effects due to exposure from pesticide cocktails have also been observed in red avadavat *Amandava amandava* (Pandey and Mohanty 2017)*.* Studies like these demonstrate the significance of assessing the risks of pesticides used in combination.

Body mass and wing length are commonly combined into a body condition index to account for differences in body size among individuals and to detect variation potentially associated with environmental stressors such as pesticide exposure. The sample size in this study meant that a reliable and biologically meaningful index could not be derived. Therefore, we analysed these biometrics separately to avoid invalid inferences. We did not detect any significant associations between biometric parameters such as age, sex, weight and wing length, and pesticide concentrations detected. Nevertheless, it remains important to test for these relationships as pesticide exposure is linked with lethal and sublethal effects on a physiological level as shown in several laboratory studies (Kitulagodage et al*.* 2011; Lopez-Antia et al*.* 2013; Mineau and Palmer, 2013; Pandey and Mohanty 2017; Rogers et al*.* 2019). The small sample size here may have reduced the power of the statistical tests conducted. For future studies, if possible, a larger sample size would be recommended to distinguish small effects from random variability. A larger sample size would also be valuable to compare pesticide profiles between sites, within species and between species within sites.

The amount of feathers that were obtained per individual also limited our ability to test and compare pesticide contamination levels internally and externally. Internal feather contamination occurs because of pesticide ingestion, which enters the bloodstream and will transfer to feathers during the growth phase. Once feathers are fully grown, these levels will be fixed at the time of growth (Jaspers et al. 2019). If we were able to do both, this comparison could depict the environmental pesticide levels or pesticide exposure levels during different periods of an individual’s life (e.g. growth phase vs. current or more recent period) from which we would identify acute vs. chronic exposure of pesticides. Differences in the pesticides detected could also identify exposure routes—as some pesticides may only be detected internally (exposure via ingestion) while others may only be detected externally (dermal contact or in nesting material) from which we can infer how birds are encountering pesticides present in the environment.

This study highlights the value of using feathers to monitor pesticide presence in the environment, including both agricultural pesticides and those used for other purposes, such as veterinary and domestic. Using generalist, abundant bird species improves the likelihood of receiving a high number of samples from ringers. Generalist species such as the blackbird could become model species for monitoring pesticide contamination of the environment, in both farmland and non-farmland habitats. Additionally, by collecting feathers in this manner, we have used a much less invasive method than drawing blood or plucking feathers. Birds often lose feathers when under stress (e.g. during the ringing process) or possibly when escaping predators, so this collection method has caused no additional stress to the bird than already experienced during ringing. This method also increases the potential samples received from bird ringers as it does not require any additional licensing or permits which would be the case when collecting blood.

It is clear that common bird species are routinely exposed to a range of different pesticides, regardless of habitat, including a range of insecticides and also herbicides. Testing for other compounds would doubtless reveal more. The critical question remaining is whether this exposure impairs fitness. We do not know how concentrations in feathers relate to exposure (which may be oral or topical), or what lethal or sublethal impacts chronic exposure to pesticide mixtures might cause. Filling these knowledge gaps is a significant challenge for future resear

## Supplementary Information

Below is the link to the electronic supplementary material.

ESM 1 (DOCX 180 KB)

## Data Availability

The data that support the findings of this study are not openly available due to reasons of sensitivity and are available from the corresponding author upon reasonable request.
