# Supplementary material for: Ubiquitous presence of pesticides in feathers of common UK birds
Source: Environ Sci Pollut Res Int. 2026 Apr 21;33(15):7059–69. doi: 10.1007/s11356-026-37677-0 (PMC13156195; doi:10.1007/s11356-026-37677-0)
Supplement: Supplementary file 1 — (DOCX 180 KB) [file 11356_2026_37677_MOESM1_ESM.docx]

**Supplementary Information: Ubiquitous presence of pesticides in feathers of common UK birds**

Priyesha Tank^a,^, Cannelle Tassin de Montaigu^a^, Gaetan Glauser^b^, Sylvie Guinchard^b^ & Dave Goulson^a^

^a^ University of Sussex School of Life Sciences, UNITED KINGDOM OF GREAT BRITAIN AND NORTHERN IRELAND

^b^ Universite de Neuchatel Faculte des Sciences, SWITZERLAND

Corresponding author: Cannelle Tassin de Montaigu,

[ct430@sussex.ac.uk](mailto:ct430@sussex.ac.uk)

University of Sussex School of Life Sciences,

Falmer, East Sussex,

UNITED KINGDOM OF GREAT BRITAIN AND NORTHERN IRELAND

Table 1. The pesticides tested for in the chemical analysis alongside their corresponding limits of quantification (LOQ) and detection (LOD) in feathers. * Variable LOQ and LOD because of strong interference in certain samples.





*Table 2. Results of ANOVA test for the mean number of pesticides present between sites and between species. SS: sum of squares, df: degrees of freedom, MS: mean sum of squares, F: F-value.*





Table 3. Results of ANOVA test for the total concentration of pesticides present between sites and between species. SS: sum of squares, df: degrees of freedom, MS: mean sum of squares, F: F-value.
